# Ethical aspects of directly observed treatment for tuberculosis: a cross-cultural comparison

**DOI:** 10.1186/1472-6939-14-25

**Published:** 2013-07-02

**Authors:** Mette Sagbakken, Jan C Frich, Gunnar A Bjune, John DH Porter

**Affiliations:** 1 Department of Nursing, Faculty of Health Sciences, Oslo and Akershus University College, PB 4, St. Olavs Plass, Oslo, 0130, Norway; 2 Department of Health Management and Health Economics, Institute of Health and Society, University of Oslo, PO Box 1089, Oslo, Blindern, 0318, Norway; 3 Department of Community Medicine, Institute of Health and Society, University of Oslo, PO Box 1089, Oslo, Blindern, 0318, Norway; 4 Department of Clinical Research/Department of Global Health and Development, London School of Hygiene and Tropical Medicine, Keppel Street, London, UK

**Keywords:** Tuberculosis, Directly Observed Treatment, Ethics, Socially Disadvantaged

## Abstract

**Background:**

Tuberculosis is a major global public health challenge, and a majority of countries have adopted a version of the global strategy to fight Tuberculosis, Directly Observed Treatment, Short Course (DOTS). Drawing on results from research in Ethiopia and Norway, the aim of this paper is to highlight and discuss ethical aspects of the practice of Directly Observed Treatment (DOT) in a cross-cultural perspective.

**Discussion:**

Research from Ethiopia and Norway demonstrates that the rigid enforcement of directly observed treatment conflicts with patient autonomy, dignity and integrity. The treatment practices, especially when imposed in its strictest forms, expose those who have Tuberculosis to extra burdens and costs. Socially disadvantaged groups, such as the homeless, those employed as day labourers and those lacking rights as employees, face the highest burdens.

**Summary:**

From an ethical standpoint, we argue that a rigid practice of directly observed treatment is difficult to justify, and that responsiveness to social determinants of Tuberculosis should become an integral part of the management of Tuberculosis.

## Background

Tuberculosis (TB) is a major public health concern with 8.8 million new cases and 1.4 million deaths globally in 2010 [1]. In the mid-1980s TB attracted increased global interest because of the growing number of cases and an alarming rise in multidrug resistant cases in the industrialized part of the world [2,3]. Although drug resistant strains had been diagnosed previously in high-endemic settings, multidrug resistance was a new problem in the industrialized world. In 1991, the World Health Organization (WHO) set two targets for national TB control programmes: to detect at least 70% of all new sputum smear-positive cases arising each year and to cure at least 85% of them [4]. Essential methods for TB diagnosis and treatment were integrated into the WHO’s new TB control strategy, Directly Observed Treatment, Short Course (DOTS). Avoiding interruption of TB treatment was considered one of the major challenges in preventing the spread of drug resistance. To prevent further development of resistance against anti-TB drugs, the extensive DOTS strategy centred on controlling each patient taking their daily dose of medication [5].

Most countries have now adopted a version of DOTS. In 2007, 86% of patients were treated successfully. This was the first time the target of 85% had been exceeded at a global level since it was set in 1991 [6]. Nevertheless, studies investigating the effect of DOTS on TB incidence, treatment completion and cure do not provide convincing evidence of the value of DOTS as the main strategy in efforts to control TB. In areas where there has been a significant decline of TB, it has been difficult to separate the effect of DOTS from the effect of general socio-economic development [7]. A study of trends in TB incidence and their determinants in 134 countries found that the incidence declined more quickly in countries that had a higher human development index, greater health expenditure, lower child mortality and improved sanitation [8]. Incidence rates declined more quickly in high-income countries with lower immigration and in countries with lower HIV infection rates. While DOTS programmes have significantly contributed to a decline in TB prevalence and TB mortality, socio-economic development is still the main reason behind the decline in TB incidence in different regions of the world [9].

Directly observed treatment (DOT) is one component of DOTS. It involves observing patients during their intake of medication. A systematic review of 11 randomized and quasi-randomized controlled trials that compared DOT conducted by a health worker, family member, or community volunteer with self-administration of treatment at home found no evidence that DOT, when compared with self-administration, had any quantitatively important effect on cure or treatment completion for TB in low-, middle- and high-income countries [10]. Further, no significant difference in clinical outcomes was found between DOT given at a clinic and DOT given by a family member or community health worker, or for DOT given by a family member compared with a community health worker [10].

The impact of DOT on patients’ autonomy has been questioned [11]. In 2004, the International Union Against Tuberculosis and Lung Disease (IUATLD) appointed an ethics advisory group to develop policies and operational guidelines based on the Helsinki Declaration [12]. In 2008, the WHO’s Ethics and Health Team established a WHO task force to address ethical issues in TB care and control programmes. In 2010, the WHO published *Guidance on Ethics of Tuberculosis Prevention, Care and Control* to guide stakeholders in implementing TB control programmes [13]. These guidelines emphasize the overarching goals of TB care and control programmes, which are:

–to achieve universal access to high-quality diagnosis and patient-centred treatment

–to reduce the human suffering and socio-economic burden associated with TB

–to protect poor and vulnerable populations from TB, TB/HIV and MDR-TB

–to support development of new tools and enable their timely and effective use

–to protect and promote human rights in TB prevention, care and control.

The guideline identifies ethical values that are important to TB care and control, such as “social justice and equity” (addressing underlying root causes and existence of inequalities in society), “common good” (meaning interventions should benefit whole communities), “respect for patient autonomy”, “participation” and “transparency” in decision-making processes, and “effectiveness” [13].

Research in Addis Ababa, Ethiopia [14,15], and in Norway [16,17], indicates that DOT gives rise to a series of ethical issues in practice. The aim of this paper is to highlight and discuss ethical aspects of the practice of DOT from a cross-cultural perspective, drawing on results from research in Ethiopia and Norway.

## Discussion

We first give an account of some frameworks commonly used to assess ethical issues in public health interventions. We then expand the ethical perspective by including a social justice framework.

### Ethical frameworks in public health

The ethical values articulated in the *Guidance on Ethics of Tuberculosis Prevention, Care and Control* reflect many of the principles incorporated in frameworks used by ethicists in assessing the justification of public health interventions. These frameworks include a loose set of moral considerations such as producing benefits, preventing harms and maximizing utility, and, to a great extent, they overlap [18-20]. Childress et al. [18] point to five “justificatory conditions” that need to be assessed when considering ethical concerns in public health interventions. These conditions are intended to help decide whether public health measures can legitimize overriding moral values such as individual liberty and justice. One condition is “effectiveness”, implying that any infringement of one or more moral considerations should be based on public, scientific evidence that the proposed intervention will do more good than harm. Another condition is “proportionality”, implying that the benefits of any proposed intervention should outweigh any negative features and effects, such as infringement of patient autonomy. Related to this is “necessity”, meaning that any intervention that raises moral concerns should not go beyond what is necessary to achieve its goal. Even when a proposed policy satisfies the first three justificatory conditions, “least infringement” is a condition underlining the need to minimize the infringement on general moral considerations. For example, when a policy infringes on a patient’s autonomy, the least restrictive alternative should be sought; or when a policy infringes on a patient’s privacy, the least intrusive alternative should be sought. Lastly, “public justification” is needed when public health agents implement practices or policies that infringe on one or several moral considerations. This justification implies the responsibility to explain and justify the necessity of the infringement in a solid and transparent way to the public [18].

Upshur [20] introduces a set of principles for public health agents to be used for systematic reflection on ethical issues in the practice of public health, and which emphasize the principles of harm, least restrictive means and transparency. These principles overlap with Childress’ five conditions, but Upshur also draws attention to the principle of reciprocity. He underlines that if burdens are imposed on a person in order to comply with a public health request, then the community should reciprocate by ensuring that such burdens, such as the use of time or income, are minimized or compensated for. Kass [19] emphasizes that even if evidence of effectiveness does exist, one must identify the known or potential burdens or harms caused by a programme. Such burdens or harms will fall into three main categories: risks related to privacy/confidentiality; risks related to liberty/self-determination; and risks related to justice (such as undue burdens imposed on particular segments of the society). One has to ask whether burdens can be minimized or if alternative approaches exist. If more than one option exists to address a public health problem, we are, according to Kass, obliged to choose the approach that represents the least risk to other moral claims, such as liberty, opportunities, privacy and justice. Health professionals thus have a responsibility to identify and advocate for the removal of health programmes that are unethical, whether because of lack of data demonstrating effectiveness, discriminatory procedures or unjustified restrictions on patients’ liberties. Kass [19] underlines the need to discuss whether public health programmes have any role in addressing existing injustices, and relates this to the strong correlation between poor living conditions and poor health outcomes.

### A social justice framework

John Rawls [21] claims that justice implies distributing resources unequally to help those that are least well off. From a social justice perspective all lives have equal value, implying a moral justification for a fair allocation of resources. Rawls’ framework requires preferential treatment for the most disadvantaged. In other words, justice would imply the use of policies that seek to safeguard the interests of all members in the community. If an uneven distribution is avoidable it may be considered unjust and unfair, and health inequalities then take on a moral and ethical dimension [13]. The social justice perspective challenges bioethics and medical ethics by claiming that central ethical topics need to be “re-socialised”. This means that market justice must be replaced by a rights-based principle of justice [22,23]. The social justice perspective may imply a criticism of ethics for not engaging in how inequalities of all kinds are linked to the same structural forces studied by anthropologists, sociologists and epidemiologists. By not engaging in how health inequalities may be prevented, ethics serves to legitimize an extravagant belief in the efficacy of medical treatment [22].

The social justice perspective draws attention to the root causes of diseases and the existence of inequalities in society [13,24]. There is increasing knowledge about how social determinants of health, or the “causes of the causes” of ill health such as childhood conditions, globalization, urbanization, living conditions and employment conditions influence all stages of TB pathogenesis, thereby creating inequities related to exposure to infection, progression to active disease, and delayed or incorrect diagnosis, as well as health outcomes. A strong association exists between TB incidence and a country’s gross domestic product per capita. A strong socio-economic gradient is also found within countries, within cities and even across households, with the poorest having the highest risk [7,9]. The higher risk of TB among the poor can most likely be explained by greater exposure to some, or many, intermediary risk factors such as HIV, under-nutrition, crowded living and working conditions, smoking, harmful use of alcohol, diabetes and indoor air pollution [7,25]. Marginalized or socially disadvantaged populations such as indigenous people, ethnic minorities, migrants and prisoners also have a higher incidence of TB [9]. Poverty and low socio-economic status correspond with poorer treatment outcomes, which may partly be explained by the poor having limited access to high-quality care. However, in most countries, TB services are integrated into existing health systems; thus it is also important to address systemic inequities in all health systems [9].

### Comparison of DOT in a low-endemic and a high-endemic country

Research in two different countries set out to explore patients’ and health personnel’s experiences with DOT. One study was conducted in 2002 in Addis Ababa, Ethiopia, where DOT was implemented in 1993 [14,15]. The other study [16,17] was conducted in 2008 in Oslo/Akershus, Norway, where DOT was implemented in 2003. Both studies were qualitative. The study from Ethiopia included in-depth interviews with 21 patients with TB (10 patients undergoing TB treatment and 11 who had interrupted treatment), four relatives and five health workers, together with two focus group discussions with an additional 11 patients. In Norway, the data were collected through in-depth interviews with 22 patients undergoing TB treatment and 20 health workers. A large proportion of the patients in both contexts were interviewed two or three times.

#### Ethiopia

In Addis Ababa, Ethiopia, all patients, without prior selective appraisal, had to attend a health care facility for DOT on a daily basis for the first two months of treatment. There was no use of community or household DOT. Many who could not afford transportation stated that they were physically exhausted because they had to travel anything from 30 minutes to two hours each day to reach the clinics. All patients, even those with advanced symptoms, were told to queue up at the same time each morning and many had to be carried or physically supported by relatives. The rigid organization of treatment reinforced many of the following emerging problems. In a country where people often lack the benefit of having a permanent job or social security benefits when ill, a serious illness such as TB can mean loss of work or the inability to work as a day labourer. One of the main problems experienced by the majority of patients in the study was loss of employment or of the opportunity to work during treatment. Many struggled to find a way to retain some level of income, but the daily clinic attendance conflicted with the possibility of registering as a day labourer in the morning or working as maids doing household chores. Some respondents were housewives, but the household finances were still affected because their husbands lost income and risked their jobs when escorting them to the clinic. In a group of 10 patients followed over a six-month period, only one patient managed to keep his (regular) job. Patients often ended up in a downward economic spiral where loss of employment and/or income occurred at a point in time where many had already exhausted all their economic resources through the diagnostic process and where they needed extra money to cover direct (transport) and indirect expenses (loss of time and income) related to daily clinic attendance. Those who struggled most to manage treatment were the socially disadvantaged groups, such as the homeless, those employed as day labourers or working in the private sector, those lacking employee or social security rights, those who had migrated to Addis Ababa for work or TB treatment, single mothers, and others lacking a social support network. Another important finding in this study was that patients experienced that they had no power to influence how their treatment was organized, even if they actively tried to do this. Many reported being threatened, punished, humiliated or treated angrily by staff for not adhering strictly to the implicit rules of the system, even though the health workers were aware of the difficulties patients faced in accessing daily treatment. There were several examples of patients being denied access to treatment after a period of treatment interruption.

While the data from Ethiopia were from 2002, the most relevant findings are supported in a recent systematic review assessing patient costs for TB treatment and care in sub-Saharan Africa [26]. The review shows that TB patients and households in sub-Saharan Africa often incur high costs when undergoing TB treatment and care. Several of the primary studies included in the review are from Ethiopia, a country where TB diagnosis and treatment is (mainly) provided by general health workers in health facilities. One study, assessing costs related to pretreatment (diagnosis) and treatment for patients with TB and/or AIDS conducted in rural, urban and peri-urban settings in Ethiopia, found that, on average, 48% of annual household income was lost because of TB treatment [27]. Pretreatment costs, such as direct costs for diagnostic tests, represented 35% of annual household income for TB patients. However, the main cost throughout treatment was reported to be loss of income. The study also reported that substantial costs were incurred because of the time and transport required to access treatment, even though 70% of patients included in the study were living within an hour’s walking distance of the clinic [27]. Some of these findings are supported by another primary study from Ethiopia [28] showing that the costs of TB diagnosis incurred by patients and escorts represent a considerable portion of their monthly income. These costs are associated with expenses related to diagnostic tests, but even more with time lost in seeking care, which causes loss of income.

#### Norway

In Oslo/Akershus, Norway, all TB patients received DOT without prior selective appraisal. The treatment was organized through the home-based nursing services and patients received DOT in their homes on a daily basis for six months. Patients and health personnel related that because of high turnover and lack of qualified personnel, those executing DOT were often unskilled and there was a lack of continuity. Health personnel were often delayed, and there was poor compliance with routines intended to ensure that the medication was delivered within a certain timeframe.

As in Ethiopia, the majority of patients experienced DOT as rigid and inflexible and felt that they had no power to influence their own situation. Men in particular described DOT as humiliating, and related this to the powerlessness they felt by having to accept a treatment that interfered with their social and working life. Patients’ immigrant status was described as a factor making people reluctant to question the treatment. In particular, asylum seekers found it difficult to express opposition because of the situation they were in. Those who did oppose DOT by asking to administer the medicines themselves experienced subtle threats, such as the involvement of authorities, including the police. Others reported how they had been denied flexible solutions such as pill dispensers, alternative DOT providers or the possibility of keeping their medication at home, so that they could take the tablets and leave for work if the nurses were late or did not come. Some patients had experienced anger and hostility from nurses because they did not have a permanent address (involuntary movement between residences conflicts with DOT). One patient reported being denied the completion of her treatment because her frequent changes of address made her “too difficult” to treat.

Those who had jobs or attended school found that the lack of flexibility in how the treatment was organized had high social costs. A majority of patients did not have a permanent job, but had temporary engagements involving shift work or work agreements made on a day-to-day basis. These conditions caused a vulnerability that participants sought to compensate for by presenting themselves as punctual but flexible shift workers, qualities that were difficult to demonstrate because of the daily DOT arrangement. Many told that they had reduced the number of shifts they were working. This because they in the period of receiving DOT (6 months) only were able to accept evening shifts due to the nurses coming to their homes within a widely defined timeframe, such as between nine and twelve o’clock in the morning.

Many also experienced an unfortunate economic situation during the diagnostic process. Those who had been engaged only in non-permanent work were often not able to claim sick leave. Several had to reduce their number of shifts gradually because of their weakened general condition, resulting in loss of income. Others described difficulties with receiving money through social services after they had been diagnosed, either because their work was not documented or because their previous income was based on day-to-day work agreements.

We found striking similarities between how DOT was experienced in Ethiopia and Norway and how the organization of treatment affects certain patient groups. The two cases raise a series of ethical issues that we will elaborate on in further detail.

### Loss of autonomy

In both countries, patients with TB were deprived of their autonomy and lacked the opportunity to influence the delivery of their own health care. Patients were never given reasons why their treatment was organized in such a way, and nor was the inherent element of force or involuntariness ever explained or justified [18]. On the contrary, in both contexts, there was a tendency that those who argued or asked about the rationale behind their treatment received incomprehensible or misleading explanations, were rejected, or even threatened. Furthermore, even though the implementation of treatment was supposed to be driven by a rigid set of rules, there was ambiguity in the discrepancy between what was required by the patients and how the treatment was provided by health personnel. Discontinuity, unpredictability and even neglect often characterized the services of those executing DOT. In cases where treatment was interrupted, patients had asked for permission to store and administer their medicines to prevent loss of employment. However, despite patients being forced to give up work, income opportunities or even treatment, health personnel “naturally” remained in possession of their medications. Many studies show that health personnel are aware that lack of individual adjustments may force patients to choose between treatment and other important needs [29]. However, as in Ethiopia and Norway, there are still many examples of health personnel responding to such needs by the use of threats, scolding and increased rigidity [29]. Studies show that health professionals commonly remain in control of patients’ treatment cards and drug supply, a practice that can be experienced as negative and dehumanizing by patients [30]. The two cases, supported by similar studies, show that those practising DOT do not seem to relate their practice to ethical considerations such as least infringement or other moral concerns, one of the justificatory conditions proposed by Childress et al. [18]. On the contrary, it appears that a rule-based approach such as DOT discourages critical thinking and sound judgment among health personnel. The main ethical challenges, therefore, may not be reflected in the principles behind DOT, but rather in how these principles are interpreted and implemented by programme planners and local health care workers.

### Burdens on the socially disadvantaged

In Ethiopia, those who were the most vulnerable and disadvantaged, such as the very poor, single mothers, and working immigrants from rural areas, were the ones who struggled most to live up to the demands of treatment. In Norway, the social costs experienced by patients were not comparable to the costs suffered by patients in Ethiopia. However, also in Norway, the most vulnerable and disadvantaged patients, such as those without permanent jobs, were those who had the greatest difficulty in adapting to DOT. This group of patients suffered disproportionate burdens in terms of indirect costs, constraints on autonomy, and violation of dignity. In high-burden countries, the average cost of receiving TB diagnosis and treatment is estimated to drain 20–40% of annual family income, a figure that increases greatly for the poorest [9]. A recent systematic review assessing patient costs for TB treatment and care in sub-Saharan Africa found TB treatment and care-related costs to be “catastrophic” for many households. The costs incurred by patients were equivalent to 10% or more of per capita income in the countries where the primary studies included in this review were carried out [26]. The review concludes that to prevent poverty as a consequence of TB treatment, policies that decrease direct and indirect costs are urgently needed. Several studies have documented that indirect costs, such as use of time and transport expenses, may have a huge impact on the ability to complete treatment, and patients may feel forced to choose between daily treatment (DOT) and daily work or other pressing duties [29].

We can use ethical principles such as ensuring the common good, effectiveness, balancing benefits and burdens, and respecting patients’ autonomy, or we can look at these cases through the concept of social justice. Either way, there are several incongruities between the guiding principles manifested in the overall aim of the TB control programme, such as “reduce human suffering and the socio-economic burden associated with TB”, “protect poor and vulnerable populations from TB, TB/HIV, and multidrug-resistant TB”; and “protect and promote human rights in TB prevention, care and control” [13], and the way the treatment system is implemented and administered in practice. There seems to be a discrepancy between the intention behind DOT and the way it is implemented in programmes and by health workers. Because of the burden imposed on the most vulnerable patients, we claim that there is an unfair distribution of benefits and burdens, and this unfair distribution may increase human suffering and the socio-economic burdens associated with TB. Several basic human rights are disregarded [31], including the right to express opinions (Human Right Article 19), the right to work and to be protected from unemployment (Human Right Article 23), the right to medical care and necessary social services (Human Right Article 25), the right not to be subjected to degrading treatment (Human Right Article 5), and the list could be extended. The undue burden imposed on socially disadvantaged groups and the many risks to other moral claims underline the need not only to scrutinize this treatment practice but also to address why there are so few critical voices to be heard. One might ask whether the responses of health personnel would have been similar across cultures if the recipients of these services did not represent socially disadvantaged groups.

### Effectiveness, evidence and least restrictive means

We lack evidence that the provision of DOT by health personnel improves cure or treatment completion rates [10] when compared with other means of treatment, and hence we question the degree to which the principle of effectiveness and the related principles of proportionality and necessity have been adequately addressed. It is difficult to see that DOT is effective or doing more good than harm when patients who interrupt treatment risk being excluded from further treatment and follow-up because they cannot adjust to the rules of the system. We also question whether the potential benefits of DOT outweigh negative features if patients are denied the opportunity to optimize their health while receiving treatment. Similarly, we ask whether a measure such as DOT is doing more harm than good in the long run, if patients lose their jobs or social positions.

In a critical review of the evidence, ethics and effectiveness of the management of TB, Verma et al. [11] emphasize the principle of effectiveness and the principle of least restrictive means. They argue that the lack of solid evidence combined with neglect of use of least restrictive means is particularly problematic when socially disadvantaged populations are targeted. When already vulnerable segments of the population are involved, the evidence should be stronger to demonstrate that the programme will attain its goals [11].

Even though there is a lack of evidence for the effectiveness of DOT, and increasing evidence that DOT creates barriers for socially disadvantaged groups, standardized DOT has been implemented without considering least restrictive means in contexts as different as Ethiopia and Norway. Least restrictive means implies that efforts are implemented according to a continuum, where self-management represents the least restrictive means and supervised treatment on a daily basis represents the other end of the spectrum of restriction [11]. Coker [2] argues that practising least restrictive means implies that “people must be allowed to fail before being condemned for that failure”. Doyal [32] has a similar point, claiming that infringement of patient autonomy can only be justified if every effort has been made to optimize the success of treatment without such infringement.

The two cases show that efforts are not being made to optimize the success of treatment without violation of patient autonomy, even in a low-endemic, high-income context such as Norway. Patients who clearly express their will to initiate and adhere to TB treatment are forced into a treatment regimen without being allowed to display their willingness and capability to adhere to treatment [2]. This dimension of DOT challenges the ethics inherent in the principles governing this treatment programme. However, again we see tension in the dynamics between the overall principles of DOT and its interpretation and implementation in local programmes and by health workers. Hall [33] underlines that even if people surrender some degree of autonomy in exchange for membership in a community, parts of their individual autonomy will still remain. A practice where patients adhere to supervised TB treatment but experience punishment for being delayed when collecting their medication is an example of how such residual autonomy is left out of the equation. A practice where patients are not allowed to keep a supply of their own medication and as a consequence experience daily disruptions in their employment is an example of how least restrictive means does not seem to be part of the ethical judgments being made by health workers.

### Transient reciprocity caused by inadequate moral involvement?

It follows from the principle of reciprocity that if persons experience a loss of autonomy to protect the community, the community should reciprocate by ensuring that they are compensated for burdens such as use of time or loss of income [20]. There is scant regard for reciprocity in practices so rigid that patients cannot combine treatment with daily work, or where they have to walk long distances to access daily treatment no matter how ill they are. The two cases demonstrate that the principle of reciprocity receives little attention, which is surprising because reciprocity should be considered not only as compensation but also as an assurance of adherence, and subsequently an element of effectiveness. One might argue that it is just to treat everyone in the same way, but a standardized programme that violates patients’ autonomy might, first of all, confirm that the patients are poor and powerless and potentially to be blamed for their own condition. It seems fair to ask whether neglect of attending to basic ethical principles is legitimized and facilitated by the receivers of the treatment representing a group without power. A related question is whether the disregard of heavy burdens imposed on socially disadvantaged patients is justified by similar power structures, or whether there is simply inadequate moral involvement. Numerous studies indicate that poor follow-up and maltreatment by health personnel is a problem [29] and TB patients’ social profile and status as “the other” might be a reason [34]. Benatar [35] suggests that concerns about those who are poor and suffer from ill health are transient, not only because they are out of sight but also because their lives may be less highly valued.

Societies may respond to a threat such as an infectious disease by declaring a “state of emergency”, with subsequent restrictions on or withdrawal of individual rights [36]. In public health discourse, socially advantaged populations (corresponding to middle and upper classes) have historically been portrayed as possessing the valued qualities of self-denial and self-efficacy. Socially disadvantaged groups (corresponding to the working and under classes) have tended to be pictured as dirty, lazy, immoral, and without the capacity to resist their inclinations [37]. Subsequently, different types of “disciplinary measures”, including coercive measures, have been directed towards the socially disadvantaged and less powerful members of society. As Lupton [37] points out, public health efforts have also been increasingly directed towards maintaining the boundaries of populations, preventing those living within the border from being infected from those coming from the outside. Thus, groups labelled as “dirty”, “contagious”; “high-risk” and “in need of extra surveillance”, have, for centuries and up to the present day, corresponded with the poor, the working class and immigrants [37].

Another interesting explanation for the lack of ethical reflection related to treatment of infectious diseases is the misperception that the questions it potentially raises are simply too easy [34]. Most people would agree that more should be done to ensure access to AIDS and TB treatment, for example. At first glance, these types of questions appear to lack the deep philosophical significance that characterizes discussions related to abortion or euthanasia, for example. However, as demonstrated in this paper, there are many complex and intertwined ethical issues that need to be addressed. The fact that diseases such as TB primarily affect those who are vulnerable because of poverty or lack of power addresses questions of philosophical significance. The strong association between infectious diseases and poverty makes TB a topic of international distributive justice, where reciprocity is one of the values with moral significance within this discussion [34,38].

### Social justice in TB control and care—a way forward

The social justice perspective draws attention to the underlying, multifaceted primary causes of disease and the existence of inequalities in society [13,24]. TB is a disease associated with poverty and systematic disadvantage among certain groups [39], and, as suggested by the social justice perspective, systematic disadvantages can only be met with systematic responses [24]. If justice is to be outcome oriented, the underlying structural causes that makes certain groups more vulnerable to TB infection need to be addressed. The Stop TB Strategy acknowledges that various social factors put certain groups at risk of developing TB. There are also recommendations on how to provide these groups with effective treatment. In the *Guidance on Ethics of Tuberculosis Prevention, Care and Control* it is stated that, because of the role of poverty and poverty-related factors in increasing the risk of TB infection and progression to active disease, “the pursuit of social justice must now become a key component of TB control” [13]. However, there are no explicit strategies about how to address the macro-level factors that make these groups vulnerable. From a social justice perspective, the fact that many TB patients are particularly vulnerable should encourage a more persistent and concrete approach towards addressing socio-economic disparities and constructing programmes and plans that would reduce, and not reinforce, some of the most evident inequities [24]. However, during the last decades, reforms in the health sector in general have been under the strong influence of global changes in macroeconomic politics. The reforms have encouraged increased use of cost sharing, payment according to input factors, and prioritizing cost-effective medical interventions [40,41]. Uncontrolled commodification of health and commercialization of health services are linked with increased medicalization of human and social relations and the growing division between the over- and under-utilization of health services by the rich and the poor. The inverse care law [42] states that the availability of good medical or social care tends to vary inversely with the needs of the population being served. As addressed earlier in this paper, it is important to recognize systemic inequities within all health systems [9].

The WHO’s Stop TB strategy [6] recognizes the importance of addressing the needs of socially disadvantaged populations. However, a uniform target-driven approach that does not seek to help patients in managing burdens related to treatment may cause extra burdens for people who are socially disadvantaged; in addition, short term benefits may not be sustainable. Our studies show that a variety of social and economic costs and other burdens change and interplay over time, from the first symptoms of TB and throughout the treatment process. Thus, locally adapted services need to focus on the individual patient and her/his life situation. To ensure that patients with TB access treatment, get cured, and remain healthy, we must address the complexity of causes and the coexisting and interacting crises that follow a TB diagnosis. This can be achieved through programmes that have a holistic and process-oriented approach.

An important aspect of this must be to include patients in an on-going dialogue about how to manage treatment and restore their health in a sustainable way. Programmes with approaches based on strategies that reduce autonomy fail to empower people so that they will gain more influence over their future health and lives. Flexibility and options in the way the treatment is organized are essential and will not only secure manageable solutions for patients, but also minimize the infringement of general moral considerations, such as violation of patients’ autonomy. Today there are several examples of programmes that are adjusted to local contexts and the needs of individual patients [29,43]. Studies show that it has become more common for DOT to be implemented flexibly, such as by using lay people in the community as DOT providers [43,44]. Examples of new ways of implementing DOT in local contexts seem to recognize the need to make the services more accessible and equitable by providing incentives or enablers [43,45]. In South Africa, DOT providers run soup kitchens for TB patients, raise money to support other activities, and conduct awareness campaigns in the community [43]. In high-income countries in particular, there are examples of DOT providers seeking to identify potential physical, social and economic barriers to TB treatment, and the way of providing treatment is negotiated with each patient. In some countries, such as the United Kingdom, DOT tends to be used only in cases where patients are considered likely to fail for different reasons, such as with those who are known alcoholics [43]. In the USA, the Harlem Family DOT Clinic is an example of a clinic that works holistically by providing consistent personal support, food, tokens and other forms of assistance, including possible referrals to a substance abuse counsellor, social worker or health educator [46].

By addressing the socio-economic situation of a patient with TB and her/his family at the point of diagnosis, we may identify barriers to treatment adherence, as well as barriers for remaining healthy. The potential roles of cash transfer and microfinance interventions in tuberculosis control have been investigated by quantifying the effects of such measures on a selected list of TB risk factors. No intervention specifically targeted TB, but a positive impact on household food security was found in eight of nine cash transfer schemes, and three of five microfinance initiatives. Improved health care access was also documented in 10 of 12 cash transfer schemes and four of five microfinance interventions [47]. Different enabler packages, such as provision of money to poor and vulnerable households and individuals, could potentially prevent patients with TB from being impoverished and help protect and build their economic, physical and human resources. In this way, we could potentially improve access to proper treatment and reduce people’s vulnerability to TB. Interventions including provision of money could be unconditional, or related to behavioural requirements such as completion of treatment or school enrolment [47]. Other measures might include the removal of user fees for TB diagnosis and transport subsidies. An earlier cross-sectional study from Haiti showed an improvement in mortality and cure rates when supplementary food and income were provided to patients undergoing TB treatment [48]. Making food or income support an integral part of TB treatment can be an important step in addressing the needs of socially disadvantaged patients. However, to be able to advance our understanding of the role of social determinants of TB, we need more studies exploring the impact of socio-economic interventions that address specific TB risk factors. Furthermore, there is a need to design studies that explore how socio-economic interventions can be addressed within the framework of treatment programmes based on the biomedical model.

In line with the ideals of social justice is the concept of *citizenship,* defined by Marshall [49] as being a “full member of society”. The concept was developed as a response to a society where the poor and otherwise socially disadvantaged groups had to receive attention and gifts from the more affluent. One of the ideas behind citizenship is that all members of the society should experience that they are true *citizens,* and hence the society as a whole has to adjust for and contribute to this*.* Being a true citizen includes having equal civil and political rights and enabling each individual to influence his or her own situation. Within the perspective of citizenship, the health professions are among those that are there to help realize the rights and opportunities of those who are socially disadvantaged [50]. However, as seen in the examples provided in this article, health personnel tend to focus more on achieving short-term goals by the use of disciplinary measures, rather than preserving citizenship*.*

## Summary

A rigid practice of directly observed treatment imposes extra burdens on already vulnerable patients, may be counterproductive, and is therefore difficult to justify from an ethical perspective. We argue that responsiveness to social determinants of TB should become an integral part of the WHO TB Control Strategy. Lessening the vulnerability of socially disadvantaged people by intervening through TB control programmes can reduce the unequal consequences of ill health and prevent further impoverishment and injustice among disadvantaged populations. Explicit and innovative strategies on how to integrate and address their needs are therefore necessary.

## Abbreviations

DOT: Directly observed treatment; DOTS: Directly observed treatment, short course; IUATLD: International union against tuberculosis and lung disease; TB: Tuberculosis; WHO: World Health Organization.

## Competing interests

The authors declare that they have no competing interests.

## Authors’ contributions

MS, JCF, GB and JDP planned the manuscript. MS drafted the first version of the manuscript, and all authors have contributed in critically revising the manuscript. All authors read and approved the final manuscript.

## Authors’ information

MS is a registered nurse and Associate Professor in the Department of Nursing, Oslo University College, and at the Institute of Health and Society, University of Oslo. JCF is a medical doctor and Professor of Health Care Management, University of Oslo. GB is a medical doctor and Professor of International Health, University of Oslo. JDP is a medical doctor and Professor of International Health, London School of Hygiene & Tropical Medicine.

## Pre-publication history

The pre-publication history for this paper can be accessed here:

http://www.biomedcentral.com/1472-6939/14/25/prepub

## References

[B1] WHO Global tuberculosis control 2011 Link: http://www.who.int/tb/publications/global_report/en/. Accessed 18.08.12

[B2] Coker R From chaos to coercion 2000 New York: St Martins Press

[B3] Ogden J Walt G Lush L The politics of 'branding' in policy transfer: the case of DOTS for tuberculosis control SocSci Med 2003 57 1 179 188 10.1016/S0277-9536(02)00373-8 12753826

[B4] Dye C Hosseini M Watt C Did we reach the 2005 targets for tuberculosis control? Bull World Health Organ 2007 85 5 364 369 10.2471/BLT.06.037580 17639221 PMC2636663

[B5] Lienhardt C Ogden JA Tuberculosis control in low-income countries: have we reached the limits of the universal paradigm? Trop Med Int Health 2004 9 7 833 841 10.1111/j.1365-3156.2004.01273.x 15228495

[B6] WHO Global tuberculosis control: A short update to the 2009 report 2009 Link: http://www.who.int/tb/publications/global_report/2009/update/en/index.html. Accessed 18.08.12

[B7] Lönnroth K Jaramillo E Williams BG Dye C Raviglione M Drivers of tuberculosis epidemics: the role of risk factors and social determinants Soc Sci Med 2009 68 12 2240 2246 10.1016/j.socscimed.2009.03.041 19394122

[B8] Dye C Lönnroth K Jaramillo E Williams BG Raviglione M Trends in tuberculosis incidence and their determinants in 134 countries Bull World Health Organ 2009 87 9 683 691 10.2471/BLT.08.058453 19784448 PMC2739916

[B9] Rasanathan K Sivasankara Kurup A Jaramillo E Lönnroth K The social determinants of health: key to global tuberculosis control Int J Tuberc Lung Dis 2011 15 Suppl 2 30 36 10.5588/ijtld.10.0691 21740657

[B10] Volmink J Garner P Directly observed therapy for treating tuberculosis Cochrane Database Syst Rev 2007 17 4 CD003343 17943789 10.1002/14651858.CD003343.pub3

[B11] Verma G Upshur RE Rea E Benatar SR Critical reflections on evidence, ethics and effectiveness in the management of tuberculosis: public health and global perspectives BMC Med Ethics 2004 5 2 10.1186/1472-6939-5-2 PMC394337 15113419

[B12] Fanning A An ethical consideration of TB: still ‘a social disease with a medical aspect'? Int J Tuberc Lung Dis 2008 12 3 229 18284823

[B13] WHO Guidance on Ethics of tuberculosis prevention, care and control Link: http://www.who.int/tb/features_archive/ethics/en/index.html. Accessed 18.08.12

[B14] Sagbakken M Frich JC Bjune G Barriers and enablers in the management of tuberculosis treatment in Addis Ababa, Ethiopia: a qualitative study BMC Public Health 2008 8 11 10.1186/1471-2458-8-11 18186946 PMC2257959

[B15] Sagbakken M Frich JC Bjune G Perception and management of tuberculosis symptoms in Addis Ababa, Ethiopia Qual Health Res 2008 18 1356 1366 10.1177/1049732308322596 18703818

[B16] Sagbakken M Bjune GA Frich JC Experiences of being diagnosed with tuberculosis among immigrants in Norway–factors associated with diagnostic delay: a qualitative study Scand J Public Health 2010 38 3 283 290 10.1177/1403494809357101 20056784

[B17] Sagbakken M Bjune GA Frich JC Humiliation or care? A qualitative study of patients' and health professionals' experiences with tuberculosis treatment in Norway Scand J Caring Sci 2012 26 2 313 323 10.1111/j.1471-6712.2011.00935.x 22043979

[B18] Childress JF Faden RR Gaare RD Gostin LO Kahn J Bonnie RJ Kass NE Mastroianni AC Moreno JD Nieburg P Public health ethics: Mapping the terrain J Law Med Ethics 2002 30 2 170 178 10.1111/j.1748-720X.2002.tb00384.x 12066595

[B19] Kass NE An ethics framework for public health Am J Public Health 2001 91 11 1776 1782 10.2105/AJPH.91.11.1776 11684600 PMC1446875

[B20] Upshur RE Principles for the justification of public health intervention Can J Public Health 2002 93 2 101 103 11968179 10.1007/BF03404547 PMC6979585

[B21] Rawls J A theory of justice 1999 Oxford: Oxford University Press

[B22] Beauchamp DE Public health as social justice Inquiry 1976 13 1 3 14 130348

[B23] Farmer P Campos NG Rethinking medical ethics: a view from below Dev World Bioeth 2004 4 1 17 41 10.1111/j.1471-8731.2004.00065.x 15086372

[B24] Gostin LO Powers M What does social justice require for the public's health? Public health ethics and policy imperatives Health Aff (Millwood) 2006 25 4 1053 1060 10.1377/hlthaff.25.4.1053 16835186

[B25] Lönnroth K Jaramillo E Williams B Dye C Raviglione M Blas E, Sivasankara Kurup A Tuberculosis: the role of risk factors and social determinants Equity, social determinants and public health programmes 2010 Geneva, Switzerland: WHO 219 241

[B26] Barter DM Agboola SO Murray MB Barnighausen T Tuberculosis and poverty: the contribution of patients costs in sub-Saharan Africa – a systematic review BMC Public Health 2012 12 980 10.1186/1471-2458-12-980 23150901 PMC3570447

[B27] Vassal A Seme A Compernolle P Meheus F Patient costs of accessing collaborative tuberculosis and human immunodeficiency virus interventions in Ethiopia Int J Tuberc Lung Dis 2010 14 5 604 610 20392354

[B28] Mesfin MM Newell JN Madeley RJ Mirzoev TN Tareke IG Kifle YT Gessessew A Walley JD Cost implications of delays to tuberculosis diagnosis among pulmonary tuberculosis patients in Ethiopia BMC Public Health 2010 10 173 10.1186/1471-2458-10-173 20353567 PMC3091545

[B29] Munro SA Lewin SA Smith HJ Engel ME Fretheim A Volmink J Patient adherence to tuberculosis treatment: a systematic review of qualitative research PLoS Med 2007 4 e238 10.1371/journal.pmed.0040238 17676945 PMC1925126

[B30] Noyes J Popay J Directly observed therapy and tuberculosis: how can a systematic review of qualitative research contribute to improving services? A qualitative meta-synthesis J Adv Nurs 2007 57 3 227 243 10.1111/j.1365-2648.2006.04092.x 17233644

[B31] United Nations Office of the high commissioner of human rights The Universal Declaration of Human Rights Link: http://www.ohchr.org/en/udhr/pages/introduction.aspx. Accessed 18.08.12

[B32] Doyal L Moral problems in the use of coercion in dealing with nonadherence in the diagnosis and treatment of tuberculosis Ann N Y Acad Sci 2001 2001 953 208 215 11795414 10.1111/j.1749-6632.2001.tb11379.x

[B33] Hall SA **Should public health respect autonomy**? J Med Ethics 1992 18 14 197 201 1460648 10.1136/jme.18.4.197 PMC1376144

[B34] Selgelid MJ Ethics and infectious disease Bioethics 2005 19 3 272 289 10.1111/j.1467-8519.2005.00441.x 16167406

[B35] Benatar SR Moral Imagination: The Missing Component in Global Health PLoS Med 2005 2 12 e400 10.1371/journal.pmed.0020400 16363910 PMC1322290

[B36] Foucault M Discipline and Punish: the Birth of the Prison 1975 New York: Random House

[B37] Lupton D The imperative of health. Public health and the regulated body 1995 London: Sage Publications

[B38] Selgelid MJ Kelly PM Sleigh A Ethical challenges in TB control in the era of XDR-TB Int J Tuberc Lung Dis 2008 12 3 231 235 18284825

[B39] Farmer P Infections and Inequalities: The Modern Plagues 1999 Berkeley, CA: University of California Press

[B40] Bond P Dor G Uneven health outcomes and political resistance under residual neoliberalism in Africa Int J Health Serv 2003 33 3 607 630 10.2190/JY59-DTCM-FBWL-RCG4 14582875

[B41] Homedes N Ugalde A Why neoliberal health reforms have failed in Latin America Health Policy 2005 71 1 83 96 10.1016/j.healthpol.2004.01.011 15563995

[B42] Hart JT The Inverse Care Law Lancet 1971 27 1 405 412 4100731 10.1016/s0140-6736(71)92410-x

[B43] Macq JC Theobald S Dick J Dembele M An exploration of the concept of directly observed treatment (DOT) for tuberculosis patients: from a uniform to a customized approach Int J Tuberc Lung Dis 2003 7 103 109 12588009

[B44] Manders AJ Banerjee A van den Borne HW Harries AD Kok GJ Salaniponi FM Can guardians supervise TB treatment as well as health workers? A study on adherence during the intensive phase Int J Tuberc Lung Dis 2001 5 838 842 11573895

[B45] Macq J Torfoss T Getahun H Patient empowerment in tuberculosis control: reflecting on past documented experiences Trop Med Int Health 2007 12 873 885 10.1111/j.1365-3156.2007.01858.x 17596255

[B46] El Saadr W Pathways to completion study 2006 Link: http://cpmcnet.columbia.edu/dept/harlemtb/innovative_programs/pathways_info.htm Accessed 21.03.13

[B47] Boccia D Hargreaves J Lönnroth K Jaramillo E Weiss J Uplekar M Porter JD Evans CA Cash transfer and microfinance interventions for tuberculosis control: review of the impact evidence and policy implications Int J Tuberc Lung Dis 2011 15 Suppl 2 37 49 10.5588/ijtld.10.0438 PMC3160484 21740658

[B48] Farmer P Robin S Ramilus SL Kim JY Tuberculosis, poverty, and "compliance": Lessons from rural Haiti Semin Respir Infect 1991 6 4 254 260 1810004

[B49] Marshall TH Citizenship and social class and other essays 1950 Cambridge: University Press

[B50] MacIntyre A After virtue: a study in moral theory 1981 London: Ducksworth

